# Deep learning versus human assessors: forensic sex estimation from three-dimensional computed tomography scans

**DOI:** 10.1038/s41598-024-81718-y

**Published:** 2024-12-03

**Authors:** Ridhwan Lye, Hang Min, Jason Dowling, Zuzana Obertová, Mohamed Estai, Nur Amelia Bachtiar, Daniel Franklin

**Affiliations:** 1 Centre for Forensic Anthropology, School of Social Sciences, The University of Western Australia, Perth, Australia; 2 CSIRO Australian e-Health Research Centre, Herston, QLD Australia; 3 South Western Clinical School, University of New South Wales, Sydney, Australia; 4 School of Human Sciences, The University of Western Australia, Perth, Australia; 5 Radiology Department, Hasanuddin University, Makassar, Indonesia

**Keywords:** Forensic anthropology, Sex estimation, Artificial intelligence, Deep learning, Convolutional neural network, Indonesia, Computer science, Skeleton

## Abstract

Cranial sex estimation often relies on visual assessments made by a forensic anthropologist following published standards. However, these methods are prone to human bias and may be less accurate when applied to populations other than those for which they were originally developed with. This study explores an automatic deep learning (DL) framework to enhance sex estimation accuracy and reduce bias. Utilising 200 cranial CT scans of Indonesian individuals, various DL network configurations were evaluated against a human observer. The most accurate DL network, which learned to estimate sex and cranial traits as an auxiliary task, achieved a classification accuracy of 97%, outperforming the human observer at 82%. Grad-CAM visualisations indicated that the DL model appears to focus on certain cranial traits, while also considering overall size and shape. This study demonstrates the potential of using DL to assist forensic anthropologists in providing more accurate and less biased estimations of skeletal sex.

##  Introduction

In forensic anthropology the estimation of skeletal sex relies on a knowledge base of evolutionary theory and biological processes that explain sex-specific differences in skeletal architecture^1^. Such processes include hormonal influences in pubertal growth trajectories, the effects of musculoskeletal loading and sexual selection^2,3^. The phenotypic expression of these differences are easily observable in the skull, and as such forensic anthropologists have derived several methods that quantify variations in these phenotypic cranial traits to estimate skeletal sex^4,5^. One of the most popular methods in modern forensic practice for morphoscopic cranial sex assessment^5^ involves the assessment of five dimorphic cranial traits, as developed by Walker^6^.

The advent of virtual anthropology facilitates new approaches for forensic anthropological practice and research. Many of the extant methods in the literature, including Walker^6^, were derived from the analysis of physical documented skeletal samples, which largely represent individuals who lived in the 19th and 20th century (see Franklin and Blau^7^, and references therein). Considering that secular variations may have a significant effect on the reliability of sex estimation methods, contemporary samples are required to validate and potentially adapt existing methods for forensic application^8–10^. For instance, the Walker^6^ method was derived from English/American and Native American population groups, and its application outside of the US reported classification accuracies that were lower^10–14^ than those in the original publication (e.g., 53.57% for females in a Greek population compared to 86.4% in Walker^6^). Sexual dimorphism in the skeleton naturally/typically varies between population groups^7^, and this variation is known to negatively affect the accuracy of sex estimation^15,16^.

The utility of clinical digital imaging, such as computed tomography (CT), enables researchers to obtain skeletal datasets that are representative of a contemporary population^7^. Virtual collections of documented skeletons or parts thereof using clinical imaging are more readily established, and arguably more representative of contemporary populations, than physical skeletal collections. As a result, morphoscopic sex estimation methods have now been validated for use with CT scans^11,15^. In addition to using virtual reference samples, machine learning has emerged as a means for handling large data sets, creating models for estimating skeletal sex, and functions to assist the forensic anthropologist in performing a biological assessment^17,18^.

While earlier studies performed skeletal sex estimation using traditional ML algorithms (e.g., random forest, support vector machines, and naïve-Bayes classifiers) with cranial traits assessed by human observers^19–23^, recent advancements in deep learning (DL) allow models to directly learn meaningful features for sex estimation without relying on predefined traits. DL models can analyse complex patterns within the imaging data (e.g., CT) that may not be immediately apparent to human observers, potentially reducing the subjectivity and biases inherent in traditional morphoscopic methods^17,18^, while offering greater adaptability when applied to different population groups. DL technology has significantly evolved and improved in recent years and has demonstrated highly accurate classification potential in skeletal sex estimation. Bewes et al.^18^ trained GoogLeNet^24^ to estimate skeletal sex from 2D lateral images of 3D reconstructions from cranial CT scans, achieving a classification accuracy of 96% for males and 94% for females. Kondou et al.^25^ utilised a DL gated attention-based multi-instance learning (MIL) model with DenseNet121^26^ for feature extraction to estimate sex from cranial CT scans and compared them against the performance of three human observers. The MIL model outperformed the human observers, achieving classification accuracies of 92% for females and 95% for males, compared to 61–88% and 65–89% for the three human observers, respectively.

Despite these advancements, previous DL-based skeletal sex estimation methods still have limitations. Bewes et al.^18^ used 2D projection images manually derived from 3D radiological images, without fully considering volumetric information. Kondou et al.^25^ employed 3D DL models for sex estimation from CT images. However, this study relied on commercial software to remove surrounding structures and extracted the skull by thresholding the Hounsfield Unit (HU) with an empirical value, which could be subject to issues such as software accessibility, noise, artifacts, unwanted bone structures, and variability in HU values. Another major limitation is explainability. Compared to cranial traits, which can be easily identified by human observers, DL networks are often referred to as the ‘black box’, wherein its hidden layers are often difficult to deconstruct, preventing human observers from identifying features deemed significant by the DL model^18^. Although estimating skeletal sex using DL models exists in the literature, there is still a lack of fully automatic and interpretable DL models for sex estimation, and a paucity of research empirically assessing their impact and potential benefits for the forensic anthropological toolkit.

Consequently, the present study aims to develop a fully automatic AI framework for forensic sex estimation using cranial CT scans and investigate the impact of incorporating the cranial traits in Walker^6^ in the model’s learning. The performance of the AI framework is then compared against that of a human observer estimating sex using the Walker^6^ standard. The AI framework consists of a pre-processing stage that uses a pretrained DL network for skull segmentation and a sex classification network. Different classification network configurations are trained with various input compositions, employing either multi-task learning to generate Walker trait scores and estimate sex, or single-task learning to estimate sex alone. To enhance the interpretability of the decision-making from the DL networks, gradient-weighted class activation mapping (Grad-CAM) is employed to visualise the discriminative cranial regions identified by the network.

## Results

###  DL network and human observer performance in sex estimation

The configurations of the DL networks with different inputs and output tasks, are outlined in Table 1. The performance of the DL network and human observer is detailed in Table 2. Among the different inputs, the network configurations which include soft tissue information captured in the CT images generally show higher AUC and comparable or improved accuracy compared to those which isolate only the skull as inputs (except for N2). Among the three network architectures, the multi-task configuration in N2, which estimates Walker trait scores and sex in separate branches, achieved the highest overall average area under the receiver operating characteristic curve (AUROC) and accuracy across different inputs, standing out as the most balanced model for sex estimation. When given the skull region $\:I\cap\:S$ as input, N2 achieved the highest accuracy of 0.97 and the lowest log loss of 0.30 compared to N1 and N3. The multi-task configuration in N1, which estimates Walker trait scores and then sex sequentially, achieved an accuracy of 0.91 when using the skull region as input, while its overall AUROC across different inputs was lower than both N2 and N3, and its log loss was higher. Although the single-task network N3, which directly estimates sex, reported similar overall AUROC to N2 across different inputs, it yielded an accuracy of 0.85 when using the skull region as input, the lowest among all DL networks.

**Table 1:** The DL network configurations used in this study and their associated outputs.

Network	Input	Loss function	Output
N1	$\:I$	$\:{L}_{comb}$	Walker trait score, sex estimate
	$$\:(I,\:S)$$		
	$$\:I\cap\:S$$		
N2	$\:I$	$\:{L}_{comb}$	Walker trait score, sex estimate
	$$\:(I,\:S)$$		
	$$\:I\cap\:S$$		
N3	$\:I$	$\:{L}_{BCE}$	Sex estimate
	$$\:(I,\:S)$$		
	$$\:I\cap\:S$$		

$\:I$ = pre-processed CT images; $\:(I,S)$ = two-channel input with pre-processed CT images and skull mask; $\:I\cap\:S$ = intersection of pre-processed CT images and skull mask; $\:{L}_{comb}$ = combined loss function; $\:{L}_{BCE}$ = binary cross entropy loss function.

**Table 2:** Performance metrics of all DL network configurations and human observer.

Observer	Variant	Input	AUROC	Acc	Sen	Spe	SB	Log loss
AI	N1	$\:I$	0.97 ± 0.02	0.97	0.93	1	0.07	0.42
		$\:(I,S)$	0.94 ± 0.05	0.91	1	0.84	–0.16	0.39
		$\:I\cap\:S$	0.89 ± 0.06	0.91	1	0.84	–0.16	0.44
	N2	$\:I$	0.99 ± 0.01	0.97	1	0.95	–0.05	0.13
		$\:(I,S)$	0.98 ± 0.02	0.94	1	0.89	–0.11	0.15
		$\:I\cap\:S$	0.93 ± 0.05	0.97	1	0.95	–0.05	0.30
	N3	$\:I$	0.98 ± 0.02	0.94	0.93	0.95	0.01	0.15
		$\:(I,S)$	0.99 ± 0.01	0.97	1	0.95	–0.05	0.14
		$\:I\cap\:S$	0.93 ± 0.05	0.85	1	0.74	–0.26	0.36
Human	–			0.82	0.93	0.74	–0.19	

AUROC = area under the ROC curve; Acc = accuracy (overall classification correctness); Sen = sensitivity (female classification accuracy); Spe = specificity (male classification accuracy); SB = sex bias (specificity – sensitivity). For DL networks, the AUROC is the average across all five models from the 5-fold cross validation. The log loss is calculated between the true labels and the average probability of being female.

When comparing the performance of the human observer to the DL network—specifically those using the skull for input, all three networks achieved a higher accuracy (N1: 0.91, N2: 0.97, N3: 0.85) in sex classification than the human observer (0.82). All networks achieved a sensitivity (female classification) of 1.0, while the human observer achieved a sensitivity of 0.93. N1 and N2 achieved specificities (male classification) of 0.84 and 0.95 respectively, both higher than the human observer (0.74). N3 had the same specificity as the human observer. Both N1 and N2 exhibited lower sex bias (N1: 0.16, N2: 0.05) compared to the human observer (0.19), while N3 had a higher sex bias (0.26).

###  Visual interpretation of network decisions using Grad-CAM

**Fig. 1:**
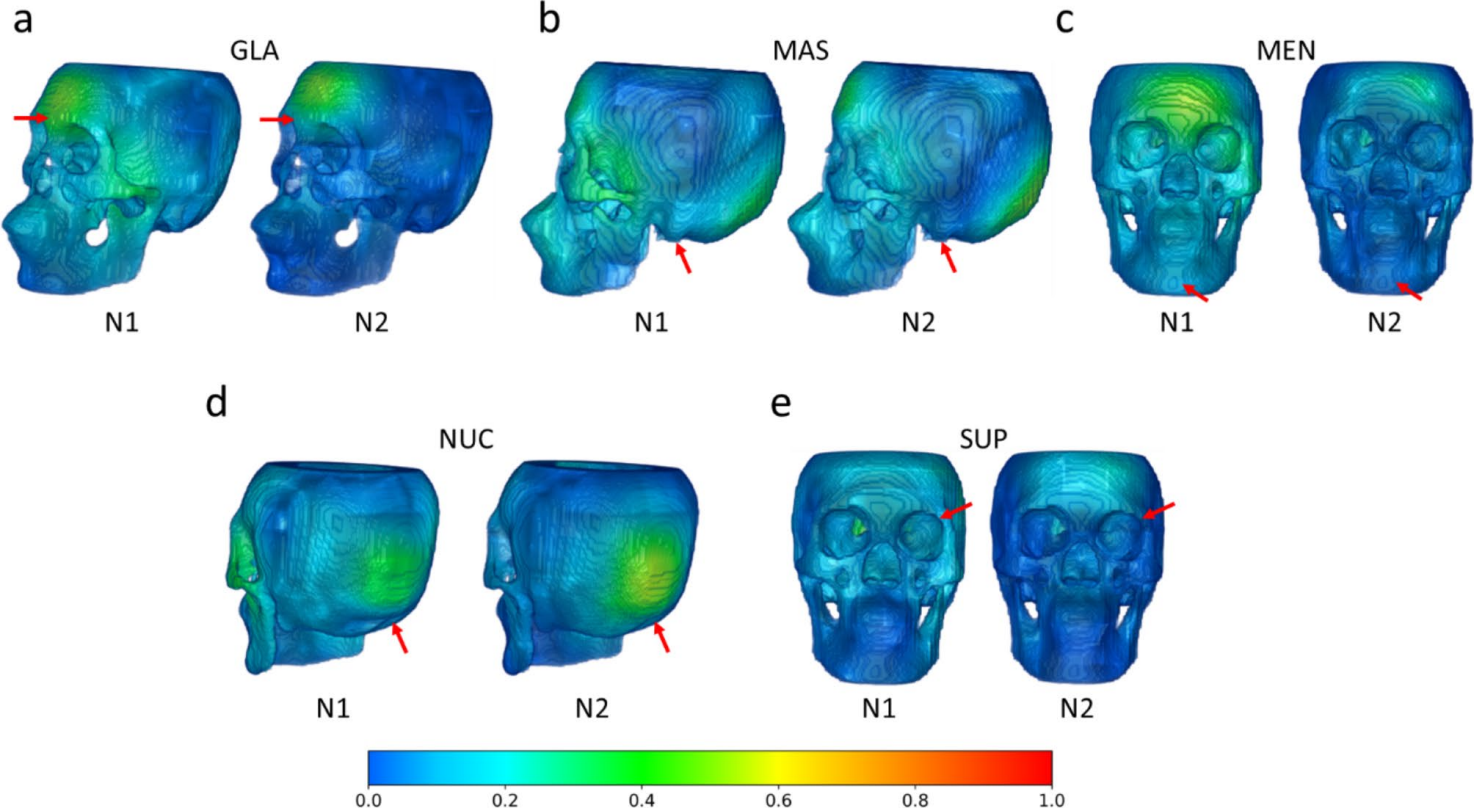
Grad-CAM heatmaps associated with each Walker score branch with input $\:I\cap\:S$, assigned by networks N1 and N2. Positions of each cranial trait is indicated with the red arrow. The heatmaps are overlayed on the skull mask and its intensity of regional activation is indicated by the colour gradient. (**a**) Glabella (GLA). (**b**) Mastoid process (MAS). (**c**) Mental eminence (MEN). (**d**) Nuchal crest (NUC). (**e**) Supraorbital margin (SUP).

To interpret the network decision making process, Grad-CAM was used to visualise the discriminative area associated with the network’s Walker trait and sex estimation outputs. Firstly, Fig. 1 visualises the Grad-CAM heatmap associated with each Walker trait prediction from the Walker trait estimation branch of network N1 and N2, using the skull as input. It can be observed that the heatmaps especially highlight two Walker traits: the glabella and the nuchal crest. However, the highlight on the Grad-CAM heatmap for each element in the predicted Walker score array did not strictly correspond to the anatomical region that the position in the array was expected to highlight. This is particularly evident in the lack of activation for the mastoid process, mental eminence, and supraorbital margin.

**Fig. 2:**
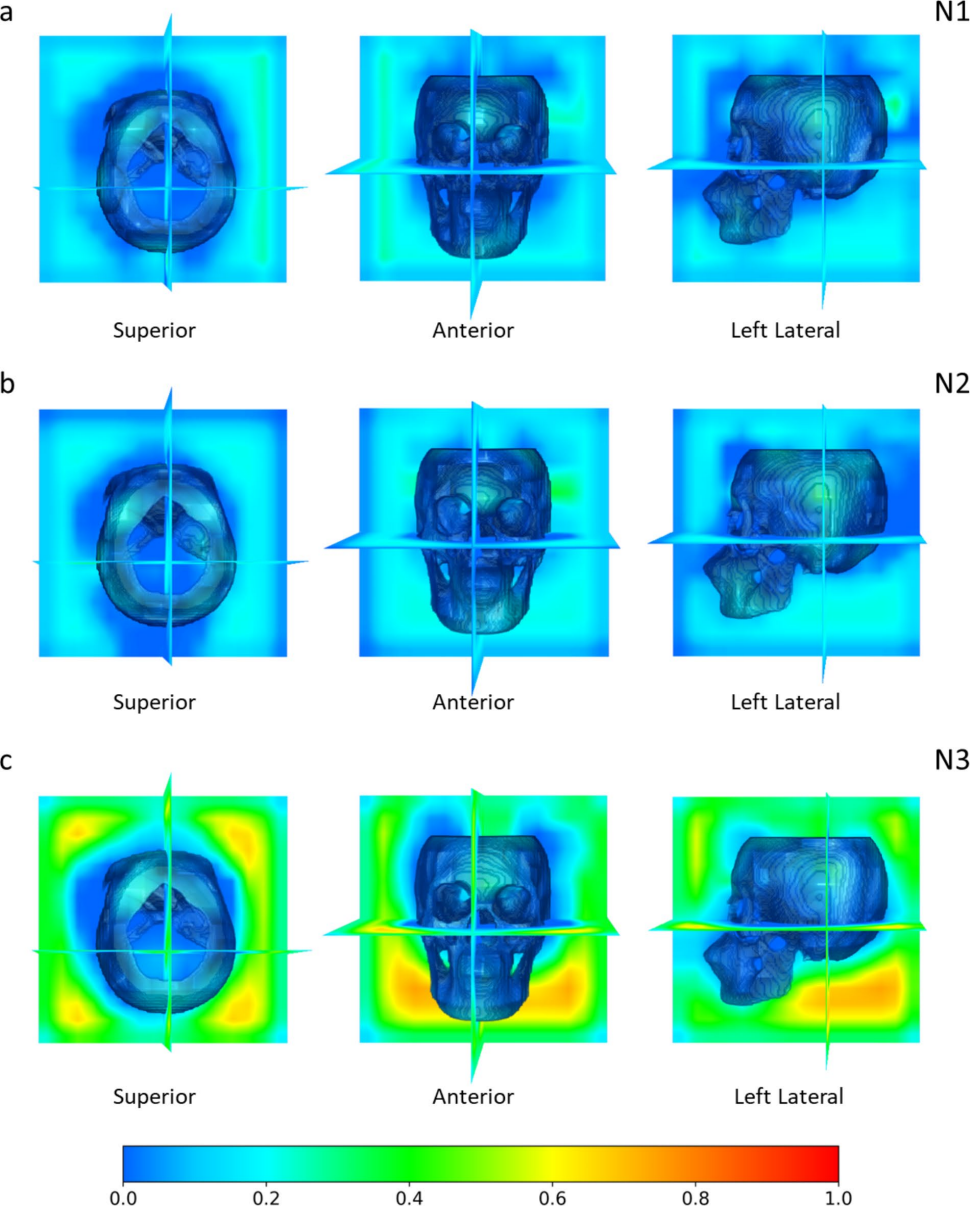
Full Grad-CAM visualisations of networks (**a**) N1, (**b**) N2, and (**c**) N3 with the $\:I\cap\:S$ input. Each network heatmap visualisation is orientated to the superior (coronal), anterior (transverse), and left lateral (sagittal) views, respectively. The intensity of regional activation is indicated by the colour gradient.

Secondly, Fig. 2 illustrates the Grad-CAM heatmaps for the sex estimation output of all three networks when using the skull as input. It can be observed that apart from a clear activation at the glabella, and less so at the mental eminence, the areas surrounding the skull are also activated, particularly in the heatmap generated for N3. Given that the CT images were pre-processed into a uniform physical size ($\:256\times\:256\times\:256\:$voxels with a voxel size of $\:1\times\:1\times\:1\:{\text{m}\text{m}}^{3}$), this could be an indication that the models were analysing the morphology of the entire skull, possibly its size and shape.

## Discussion

The aim of the present study was to develop a fully automatic DL framework for the estimation of skeletal sex, with the inclusion of Grad-CAM heatmaps to identify features the DL network used for interpretation. Multiple configurations of the network with different inputs were explored to assess whether incorporating Walker trait scores in the models’ learning would improve sex estimation performance. Additionally, the performance of the DL networks was compared to that of a human observer. Three key findings were made in this study. Firstly, the multi-task DL network, which estimated sex and Walker trait scores as an auxiliary task, achieved the highest accuracy (0.97) in sex estimation when using the skull as input. Secondly, the Grad-CAM visualisations suggested the DL network estimated sex by considering some of the Walker traits and potentially the overall morphology of the skull. Lastly, all DL networks reported higher accuracies for sex estimation when compared against the human observer using the five Walker traits on the test dataset.

Three different types of input were explored in this study, including the whole CT image $\:I$, CT image and skull mask in two channels $\:\left(I,\:S\right)$ and skull region alone $\:I\cap\:S$. The inclusion of images with soft tissue improved sex estimation AUROC across all three networks, and generally improved or maintained accuracy, except for N2, when compared against the skull region alone. It is important to note that the CT scans analysed in this study were obtained from living individuals, and therefore include soft tissue information. Forensic anthropologists specialise in osteological examination and do not consider soft tissue information. As such, the performance of DL networks that use the skull ($\:I\cap\:S$) as input is considered a representative comparison to that of a human observer.

When given the skull region $\:I\cap\:S$ as the input, N2 achieved the highest accuracy and average AUROC. Although N3 achieved the same AUROC as N2, its accuracy on the test set was lower than that of N2 and N1 when the operating point was selected based on validation performance. This discrepancy indicates that the auxiliary task of estimating Walker trait scores in N2 and N1 is beneficial, improving the ability for the DL network to generalise better to unseen data. When comparing the performance of the DL networks against the human observer using BLR, the DL networks, regardless of input, were more accurate on the test set. All three DL network configurations achieved a higher accuracy than the human observer using BLR with Walker traits. Specifically, N2, when utilising only the skull region $\:I\cap\:S$ as input, achieved the highest sex estimation accuracy of 97%, compared to 82% for the human observer. In addition to the BLR model used by the human observer, as applied in the original Walker^6^ study, machine learning models such as support vector machine (SVM) and random forest were also implemented using human-generated Walker traits to estimate sex. The SVM achieved results comparable to BLR in terms of accuracy, sensitivity, and specificity, while the random forest model performed better, with an accuracy of 0.85, sensitivity of 0.93, and specificity of 0.79.

Both the human observer using Walker traits and the DL networks using the skull as input showed a bias towards misclassifying males in the Indonesian dataset. However, the most accurate performing DL network achieved a lower sex bias (0.05) compared to the human observer (0.19). Rogers et al.^27^ tested the Walker^6^ standard in an Indian population and reported male classification accuracy as low as 21.05%. Similar performance was also noted when the Walker standard was applied to a Thai and Japanese population (30.2% and 34.9%, respectively)^10^. These studies accord with others in the literature in emphasising the development and use of population-specific models for sex estimation^10–14^. The implementation of AI models into the workflow may serve to further improve classification accuracy and adaptability of morphoscopic methods in forensic anthropology.

The Grad-CAM heatmaps visualised the discriminative regions of the skull the DL networks focused on for Walker trait scores and sex estimation. For Walker trait score estimation with N1 and N2, the glabella and nuchal crest were mainly highlighted, as shown in Fig. 1. However, these heatmaps also suggest that the DL networks did not isolate and assess each anatomical region for its respective traits in the same manner as a human observer. This could be because the DL networks were trained solely to replicate the numeric values associated with Walker scoring, lacking direct linkage to the physical anatomical regions corresponding to these scores. Additionally, the Walker traits and sex estimation were optimised in a combined loss function, where the two tasks could potentially interfere with each other.

In relation to Grad-CAM visualisations for the sex estimation output, the glabella and less clearly the mental eminence, were activated. Additionally, it was observed that the models potentially considered the overall shape and size of the skull, as shown by activation of regions surrounding the skull. This effect is especially prominent in N3, which was tasked with directly estimating sex. The inclusion of the general morphology of the skull in the networks is likely one of the contributing factors to the enhanced performance over the human observer who was restricted to the Walker traits in this study. The general size and shape of the skull are key features that reflect sexual dimorphism in human populations, with male skulls being overall larger and more robust than females^1,2,28^.

It is important to note that the present study has several limitations. Firstly, the dataset used is relatively small (*n* = 200) compared to other research, such as Bewes et al.^18^ and Kondou et al.^25^, which included samples of 1,000 and 2,041 cranial CT scans, respectively. However, as the dataset was obtained from Indonesia, a country with limited forensic anthropological research^11,29^, its use in this study is of considerable benefit to practitioners in both its novelty and uniqueness. Future work will aim to include datasets from diverse population groups to further refine the DL networks tested in this study. This inclusion is necessary to enhance the robustness and generalisability of the AI model, providing forensic anthropologists with a reliable tool for sex estimation of individuals whose population affinity is unknown. Secondly, it remains challenging to isolate and quantify the specific impact of certain Walker traits and other factors like skull shape and size on the model’s sex estimation. Future research will aim to conduct a more detailed interpretability analysis to gain clearer insights into how different features impact model predictions. Lastly, the pre-trained TotalSegmentator^30^ model used for skull segmentation had difficulties with the mastoid process due to variable bone density. This could compromise sex estimation accuracy based on the skull region. Future studies will aim to fine-tune the segmentation model to achieve more accurate skull segmentation.

The present study has introduced and evaluated the performance of a fully automated AI framework for sex estimation on a dataset of Indonesian individuals, with obvious broader forensic applicability relative to the underlying approach that has been developed. In general, the DL networks outperformed the human observer in the overall accuracy of sex estimation, with the best-performing model being the multi-task model that learned to estimate both Walker trait scores and sex when given the skull as input. For Walker trait score estimation, the Grad-CAM heatmaps of the multi-task models showed a focus predominantly on two traits, the glabella and nuchal crest. For sex estimation, the Grad-CAM highlighted the glabella and mental eminence, which correspond to Walker traits, but also showed much broader engagement with cranial shape and size. This focus possibly reflects the natural sexual dimorphism in human populations, with larger and more robust skulls observed in males compared to females.

This study makes a significant contribution to forensic anthropology by demonstrating the effectiveness of AI in improving the accuracy of the estimation of skeletal sex, particularly in population groups underrepresented in research. There is a benefit to both forensic anthropology and machine learning, as AI models can continuously improve with larger and more diverse data, ensuring their robustness and applicability across different population groups. AI technology, with its ability to process and analyse large datasets quickly and accurately, can be integrated into forensic practice to assist anthropologists in estimating sex and other variables of the biological profile not only in mass death situations but also in routine casework. This integration can lead to more standardised and objective assessments, reducing the impact of human bias and variability.

## Methods

###  Dataset

The sample (i.e., dataset) used in this study was obtained through the Picture Archiving and Communication Systems (PACS) database in Dr Wahidin Sudirohusodo General Hospital (RSWS) at Hasanuddin University, Indonesia. Multi-slice CT (MSCT) scans from individuals who presented at RSWS for radiological examination as part of their routine treatment between January 2020 and August 2022 were obtained. A Siemens Healthineers SOMATOM go.Top 128-slice acquired scans with in-plane resolutions from 0.26 to 0.60 mm, and slice thickness ranging from 0.45 to 1.50 mm (58.0% of all scans are 1.0 mm). The regions of interest captured in these scans include the cranium, with some scans extending to include sections of the upper torso. However, the cranial region was isolated for scoring and analysis. Scans that displayed signs of pathology, abnormalities, or any surgical modifications that obscured the visualisation or otherwise manipulate the scoring of any Walker trait were excluded at the sample collection stage.

In total, 200 MSCT scans, all including the cranial region, were obtained, and analysed in this study, comprising 87 female and 113 male individuals. Their ages at the time of examination were 15 to 76 years (female: mean age 43.6 years, SD 13.4 years; male: mean age 40.8 years, SD 15.2 years). All other patient metadata were anonymised in PACS prior to receipt. Although it cannot be fully ascertained that all individuals in the dataset are of Indonesian origin, it is assumed that patients presenting to RSWS are primarily local to the city of Makassar, and in turn Indonesians.

Permission to study the cranial CT sample was approved by the Office of the Director-General of Health Sciences from the Ministry of Health, Republic of Indonesia, through Hasanuddin University (LB.02.01/2.2/6807/2022). Ethics clearance to undertake this research was obtained by the Human Ethics division of the Office of Research at the University of Western Australia (2021/ET000377) and the Health and Medical Human Research Ethics Committee from the Commonwealth Scientific Innovation and Research Organisation (CSIRO; 2023/001/RR). As this is a retrospective study, informed consent was not necessary, in line with legislation set out in the *Privacy Act 1988 (Cth)*. All methods presented in the following subsections were performed in accordance with the Australian National Statement on Ethical Conduct in Human Research.

### 3D visualisation and sex estimation by a human observer

The dataset was visualised in OsiriX^®^ v13.0.1. The ‘3D volume rendering’ function was used for visualisation. Each scan was orientated for scoring using the ‘pan’ and ‘3D rotate’ functions. To isolate bone, the ‘high contrast’ 3D preset was used. CLUT was set to ‘VR Muscles-Bones’ and no convolution filters were applied.

**Fig. 3:**
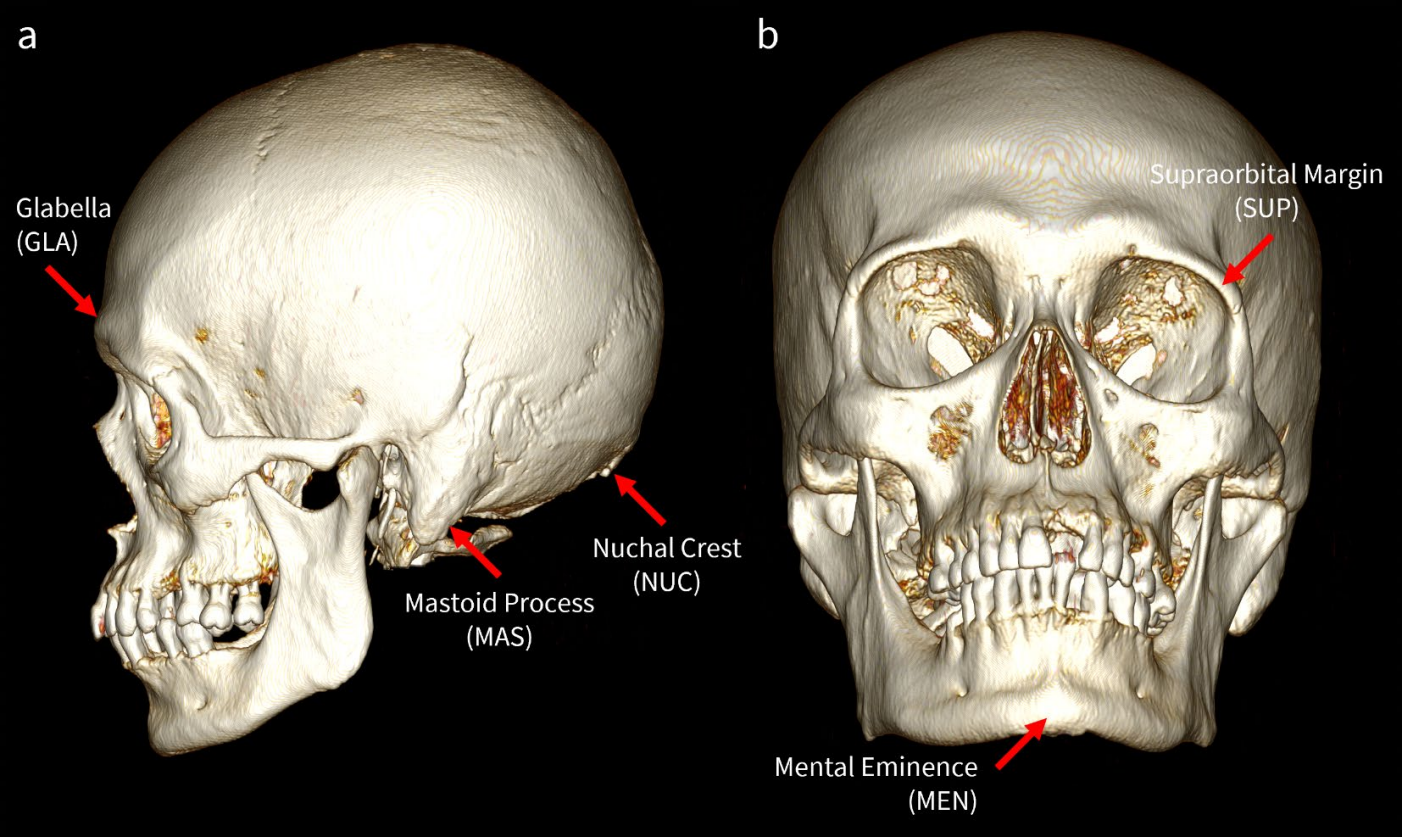
Volume-rendered CT scan visualised in OsiriX^®^, featuring the five cranial traits used in this study. (**a**) Left lateral view, with the glabella (GLA), mastoid process (MAS), and nuchal crest (NUC) indicated. (**b**) Inferio-anterior view, with the mental eminence (MEN) and supraorbital margin (SUP) indicated.

Scoring by the human observer (RL) followed descriptions and illustrations in Walker^6^. The five Walker traits featured are the glabella (GLA), mastoid process (MAS), mental eminence (MEN), nuchal crest (NUC), and supraorbital margin (SUP), all of which are visualised in the 3D environment as shown in Fig. 3. The mastoid process and supraorbital margin are bilateral traits, with scores recorded on both the left and right sides. Scores were assigned on a 5-point ordinal scale, from 1 indicating ‘minimal expression’ to 5 indicating ‘maximal expression.’ In general, higher trait scores are more likely to be associated with an individual of male sex and vice versa. However, the scores themselves are not assumed to be associated with a feminine or masculine trait expression.

Once all crania were scored, the dataset was partitioned. Five-sixth of the dataset (*n* = 166) was randomly allocated as a training subset to create a five-trait binary logistic regression (BLR) function. The remaining one-sixth was used for model testing (*n* = 34). Only scores recorded from the left of both bilateral traits are used, in line with existing literature^6,11,15^. With all Walker traits available for scoring, the five-trait BLR function for the Indonesian population is provided below:

1 $$\:{Y}_{H}=1.321\left(\text{G}\text{L}\text{A}\right)+1.092\left(\text{M}\text{A}\text{S}\right)+0.643\left(\text{M}\text{E}\text{N}\right)+0.483\left(\text{N}\text{U}\text{C}\right)+0.282\left(\text{S}\text{U}\text{P}\right)-9.800$$

where the composite score $\:{Y}_{H}$ is calculated from each Walker trait score input into Eq. 1, and is associated with a probability value $\:{p}_{H}$, derived from the following equation:

2 $$\:{p}_{H}=\frac{1}{1+{e}^{{Y}_{H}}}$$

The probability threshold for sex estimation using BLR is 0.50. $\:{p}_{H}$ values greater than the threshold are classified as female, while $\:{p}_{H}$ values less than the threshold are classified as male.

###  Sex estimation by the DL network

**Fig. 4:**
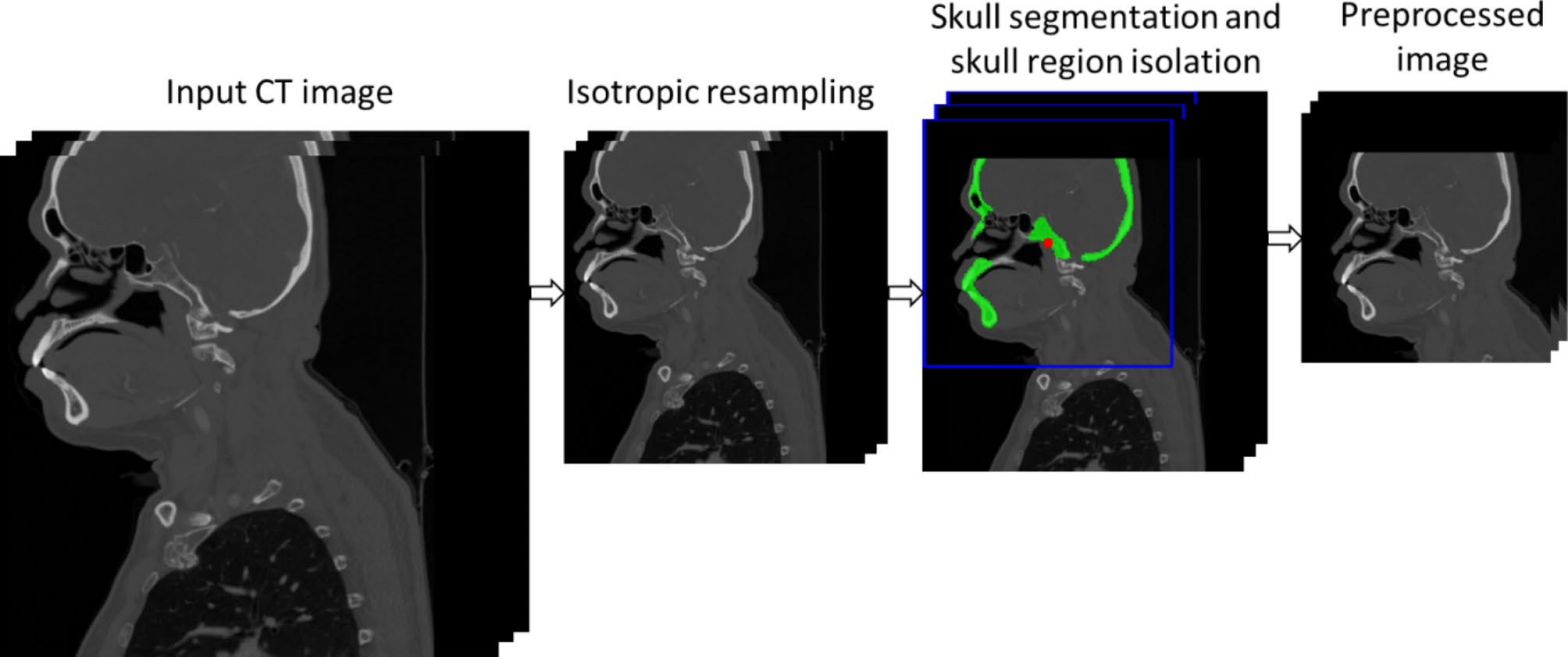
CT image pre-processing workflow: isotropic resampling, isolation of the skull using TotalSegmentator (in green) through bounding boxes (in blue) with its associated centroid (in red), and final cropped CT images using the bounding box as a boundary.

The pre-processing stage is outlined in Fig. 4. The CT images were first resampled isotopically to a spatial resolution of $\:1\times\:1\times\:1\:{\text{m}\text{m}}^{3}$. The skull was then identified using TotalSegmentator^30^, an open-source CT anatomic structure segmentation DL network. Following segmentation, the CT images were cropped into a bounding box volume of $\:256\times\:256\times\:256\:$voxels to isolate the skull from the rest of upper torso, which were included in many of the CT images. The cropping dimensions were selected to be sufficiently large to cover the entire skull. The same dataset partitioning for the creation and testing of the BLR function (Eq. 1) was used for training and testing the DL networks.

**Fig. 5:**
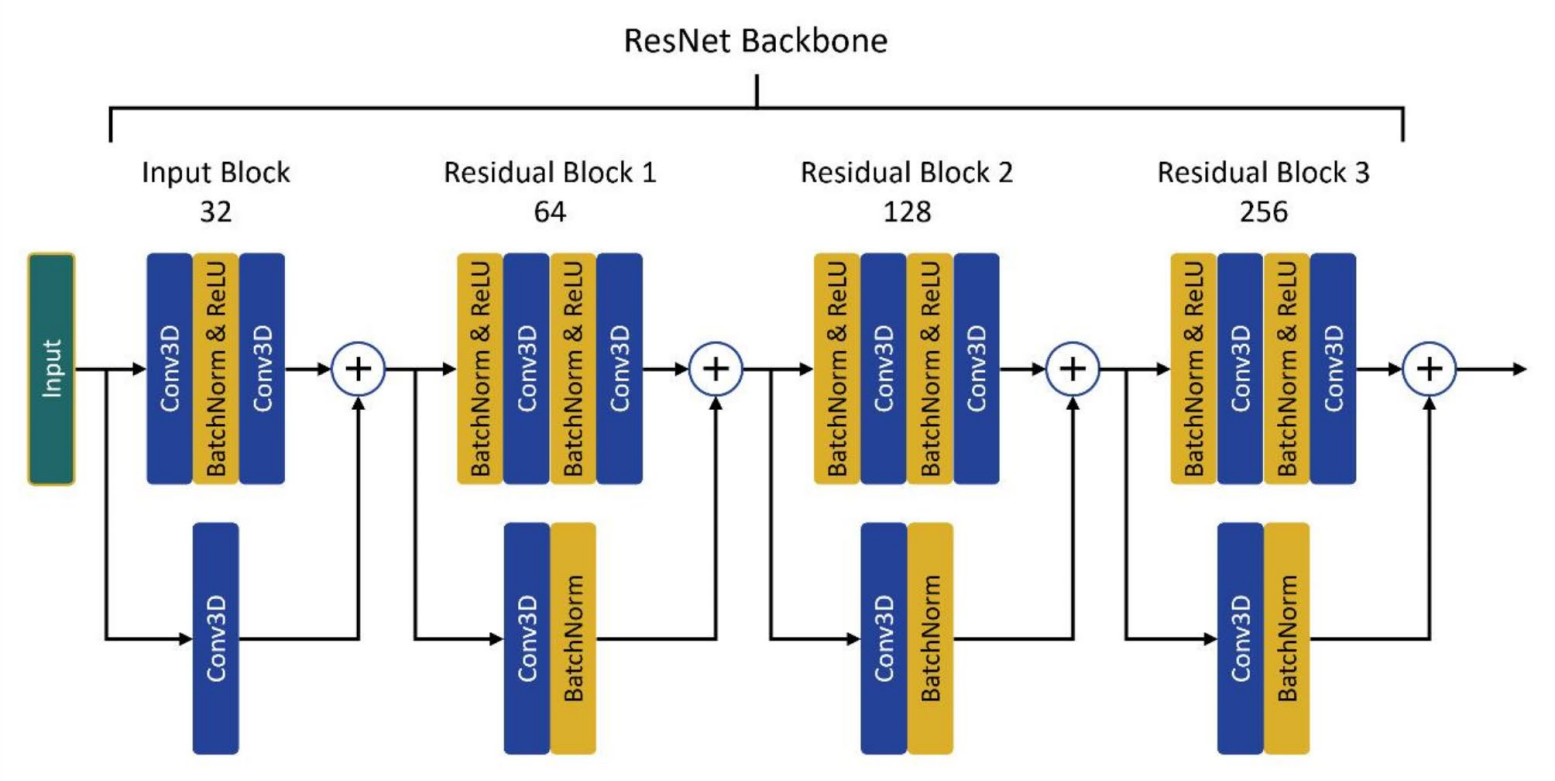
The ResNet backbone used in this study. Numbers associated with each block indicate the number of filters used.

**Fig. 6:**
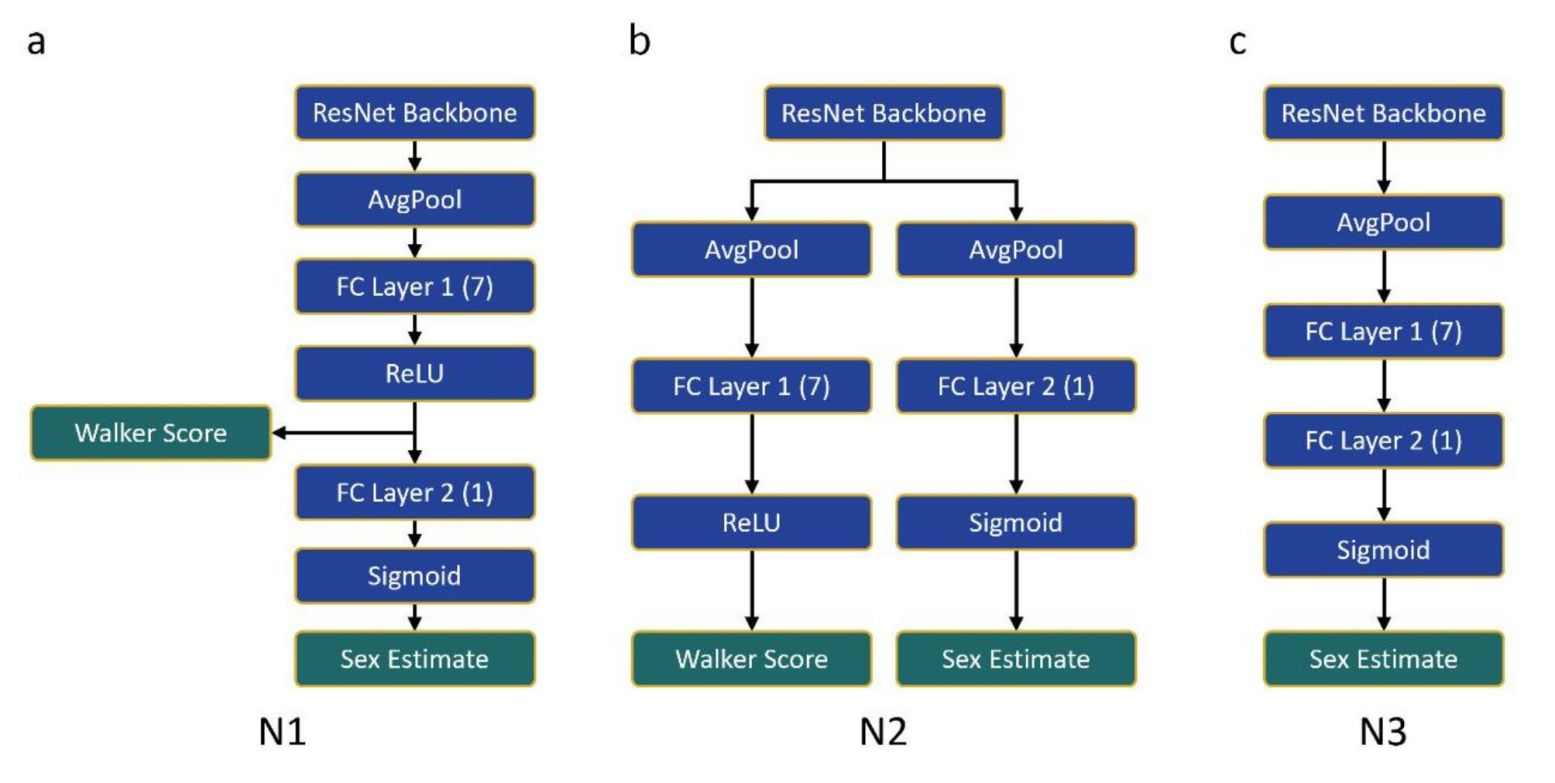
The three network architecture variants built from a ResNet backbone. The numbers in parentheses indicate the size of each fully connected (FC) layer. (**a**) N1 utilises the assigned Walker trait scores in its sex estimate output. (**b**) N2 assigns Walker trait scores and estimates sex in parallel branches. (**c**) N3 estimates sex without assigning Walker trait scores.

Three DL classification network variants, N1, N2, and N3, were built upon a ResNet backbone. As illustrated in Fig. 5, the ResNet consists of an input block and three residual blocks, which include 3D convolution (Conv3D), batch normalisation (Batch Norm), and rectified linear unit (ReLU) activation layers. The input block has 32 filters, while the three residual blocks have 64, 128, and 256 filters. The kernel size for Conv3D is $\:3\times\:3\times\:3$. The network variants and their varying output branches were constructed using this backbone as shown in Fig. 6.

Network variants N1 and N2 (Fig. 6a,b) are multi-task networks designed to generate Walker trait scores and estimate sex (probability of being female, see below) as outputs. N1 first assigns Walker trait scores through FC layer 1, which generates an array of seven numeric values, each corresponding to a Walker trait, including bilateral ones. These scores are then used to estimate sex. In contrast, N2 features two distinct output branches: one for generating Walker trait scores with an array of seven numeric values, and the other for sex estimation. Both N1 and N2 use a combined loss function ($\:{L}_{comb}$) of mean squared error (MSE) and binary cross entropy (BCE) as shown in Eq. 3:

3 $$\:{L}_{comb}={L}_{MSE}+{L}_{BCE}$$

The MSE loss function, $\:{L}_{MSE}$, is calculated using the following equation:

4 $$\:L_{{MSE}} = \frac{1}{N}\sum\limits_{{i = 1}}^{N} {(w_{i} - \hat{w}_{i} )^{2} }$$

where $\:w$ and $\:\widehat{w}$ are the Walker trait scores assigned for an individual $\:i$ by the human observer and the DL network respectively.

The BCE loss function, $\:{L}_{BCE}$, is calculated using the following equation:

5 $$\:{L}_{BCE}=-\frac{1}{N}\sum\limits_{i=1}^{N}[{y}_{i}\cdot\:\text{log}\left({p}_{i}\right)+(1-{y}_{i})\cdot\:\text{l}\text{o}\text{g}(1-{p}_{i}\left)\right]$$

where $\:y$ is recorded sex, coded as 1 for female and 0 for male, and $\:p$ is the probability of the individual $\:i$ being classified as female. N3, on the other hand, does not generate Walker trait scores and directly outputs estimated sex using the BCE loss function alone (Eq. 5).

**Fig. 7:**
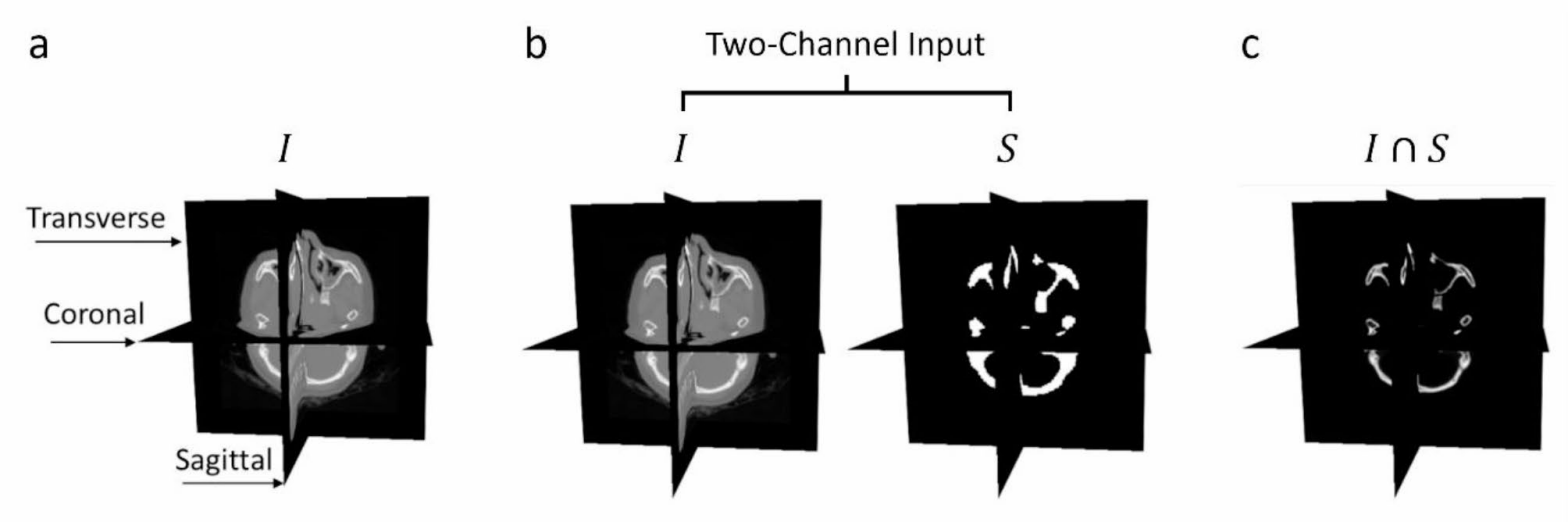
The three network input variants with major anatomical planes indicated. (**a**) Input $\:I$ – pre-processed CT images. (**b**) Input $\:(I,S)$ – a two-channel input which includes the pre-processed CT image $\:I$ and binary skull mask $\:S$ generated by TotalSegmentator. (**c**) Input $\:I\cap\:S$ – the intersection between the image $\:I$ and the skull mask $\:S$.

Three types of inputs were investigated for their impact on the sex estimation performance: the pre-processed CT image $\:I$; the image and skull mask as two channel input $\:(I,\:S)$; and the intersection between the image and the skull mask $\:I\cap\:S$. Here, $\:S$ is the skull mask generated by TotalSegmentator^30^, processed by a morphological closing with a $\:5\times\:5\times\:5$ kernel radius. These input types are outlined in Fig. 7.

To reduce computation complexity, the inputs to the networks were resized to $\:[128\times\:128\times\:128]$. During network training, data augmentation techniques, including horizontal flipping and rotation along the transverse plane were implemented. The combination of network variants, inputs, loss functions, and outputs are outlined in Table 1. Under each configuration, the network was trained within a 5-fold cross validation using the training dataset at a maximum epoch number of 100 with the Adam optimiser. In each cross-validation iteration, the model achieving the lowest loss value on the validation dataset was saved, resulting in five models per configuration. All networks were implemented in *Torch2.0* using *Python v3.9* and trained on a NVidia Tesla P100 GPU with 16GB of RAM.

For each network configuration, the five models trained through the 5-fold cross validation were applied to the testing dataset to either generate Walker trait scores and estimate sex (N1 and N2) or estimate sex alone (N3). The probability threshold for sex estimation was determined using the following equation:

6 $$\:t=\underset{t\in\:\left[\text{0,1}\right]}{\text{argmin}}\left[\text{D}\left(t\right)\right]$$

where $\:t$ is the operating point threshold that minimises the distance to the point $\:(0,\:1)$ on the ROC curve^31^, as described in Eq. 7, based on the validation data.

7 $$\:\text{D}\left(t\right)=\sqrt{{(1-\text{s}\text{e}\text{n}\text{s}\text{i}\text{t}\text{i}\text{v}\text{i}\text{t}\text{y}(t\left)\right)}^{2}+{(1-\text{s}\text{p}\text{e}\text{c}\text{i}\text{f}\text{i}\text{c}\text{i}\text{t}\text{y}(t\left)\right)}^{2}}$$

The final sex estimation from the testing dataset is derived using a majority voting mechanism across all five models. In cases where the models also generate Walker trait scores, the final score is calculated by averaging the outputs from all five models and rounding it to its closest ordinal value (i.e., 1 to 5). Grad-CAM was used to visualise and interpret the decision-making process of each model. It leverages the gradients of a specified target output flowing into the last convolutional layer. This method generates a coarse localisation map that emphasises the discriminative areas responsible for the estimation in each DL network.

### Statistical analysis of human/network performance

Performance of the DL networks and human observer in sex estimation was evaluated through six different metrics. The first is AUROC, which calculates the overall discriminative ability of a classifier over various probability thresholds for sex estimation. Second is accuracy, reflecting the overall correctness of classification. The third and fourth metrics are $\:\text{S}\text{e}\text{n}\text{s}\text{i}\text{t}\text{i}\text{v}\text{i}\text{t}\text{y}=\frac{TP}{TP+FN}$, which quantifies female classification accuracy, and $\:\text{S}\text{p}\text{e}\text{c}\text{i}\text{f}\text{i}\text{c}\text{i}\text{t}\text{y}=\frac{TN}{TN+FP}$, which reflects classification accuracy for males. Here, $\:TP$, $\:FP$, $\:TN$, and $\:FN$ stand for true positive, false positive, true negative and false negative respectively, with female as the positive class. The fifth evaluation metric is sex bias ($\:SB=\text{s}\text{p}\text{e}\text{c}\text{i}\text{f}\text{i}\text{c}\text{i}\text{t}\text{y}-\text{s}\text{e}\text{n}\text{s}\text{i}\text{t}\text{i}\text{v}\text{i}\text{t}\text{y}$), which quantifies the difference between specificity and sensitivity. A positive sex bias suggests a tendency for the model to misclassify females, while a negative sex bias suggest a tendency for the model to misclassify males. The last metric is log loss, which is equivalent to BCE loss in this case. Here, it is calculated between the true sex labels and the average probability of being female.

## Data Availability

Sample skull data, which are extracted from the CT images, and code are available at https://github.com/aehrc/ForensicSexEstimation.
